# Sex Robots—A Harbinger for Emerging AI Risk

**DOI:** 10.3389/frai.2019.00027

**Published:** 2019-11-29

**Authors:** S. E. Galaitsi, Christine Ogilvie Hendren, Benjamin Trump, Igor Linkov

**Affiliations:** ^1^Credere Associates, Westbrook, ME, United States; ^2^Department of Civil & Environmental Engineering, Duke University, Durham, NC, United States; ^3^University of Lisbon, Lisbon, Portugal; ^4^US Army Corps of Engineers, Concord, MA, United States; ^5^Carnegie Mellon University, Pittsburgh, PA, United States

**Keywords:** artificial intelligence, sex robots, risk, policy, public health

## Introduction

Artificial intelligence (AI) is becoming more commonplace in all aspects of the developed world, where robots might help with tasks as diverse as hospital operations to defusing explosives (Weng et al., 2009; Linkov et al., 2018; Murphy, 2019). Historically, the sex industry has been on the forefront of implementation of innovative technologies, and integrating AI is unlikely to be an exception. Some AI applications already seek to mimic human dynamics (Weng et al., 2009), such as the conversational Alexa or Siri, which use machine-learning technology to better predict, understand, and correctly fulfill our informational requests. However, the sex industry is innovating AI to fulfill human emotional and physical demands. Though manufacturers of Alexa decided to program the software to refrain from engaging with questions of a sexually explicit or harassing nature (Crum, 2018), digital assistant developers have reported that at least 5% of user interactions were unambiguously sexually explicit (Samuel, 2019). AI embedded in sex dolls can be specifically geared to meet this demand. Current sex robot capabilities include touch and movement detection, blinking, brow movements, head turning and tilting, and holding conversations (Realbotix, 2014; Mlot, 2018) These conversations include simple processes like remembering facts, and more complex ones like engaging with their partners' emotions (Shen, 2019).

Sex robots are complex systems where integration of AI and traditional (e.g., chemicals) and novel technologies (e.g., advanced materials) may result in widely unknown and unpredictable risks. Researchers have already noted the lack of data about public health aspects of sex robot use (Cox-George and Bewley, 2018), and concerns include addiction, social isolation, non-consensual replication of real people, and enabling misogyny, racism, and pedophilia, though supporters have also claimed that sex robots could provide safe outlets for harmful urges (see Morin, 2016; Readhead, 2016; Richardson, 2016; Maras and Shapiro, 2017; Sharkey et al., 2017; Campaign Against Sex Robots, 2018). However, risks from sex robots may also arise from the technology developed for them. Herein, we consider the pathway for risks to emerge from AI advancement within sex robots and argue that an aversion to regulating pleasure devices, combined with an aptitude for sex to spur technological innovation may produce unique risks (Levy, 2009; West, 2018). AI development in sex robots requires immediate policy attention.

## Sex and Technological Innovation

Sex sells, and emerging technologies can benefit by providing new accessibility to pleasure and taboo subjects. Pietro Aretino's pornographic *Postures* (1524) and Francois Rabelais' obscene *Gargantua and Pantagruel* (1530-40) boosted the early printing press (Zimmer and Hunter, 2003). The vibrator, patented in the early 1880s, was one of the first five appliances to be electrified in order to relieve the burden from male doctors who had been manually curing “hysteria” in their female patients, although at the time this was not acknowledged as a sexual activity.

The benefits of leveraging sex to drive emerging technology are particularly pronounced when products are intended for private consumption; for example, the VCR benefitted from an early surge in X-rated prerecorded tapes, which constituted half of all video sales in the late 1970s (Coopersmith, 2000). By the mid-1980s, the demand for pornography had not diminished, but the rest of the film industry surpassed it in production. The early camcorder industry was similarly bolstered by the market for visual sex. Photography, paperback books, modern video conferencing, and even answering machines received early support from sex media consumers before the technologies went more mainstream. In perhaps its ultimate coup, pornography also helped establish the market for the internet (Coopersmith, 2000). Should a similar development trajectory be anticipated for sex robots, wherein they will shed their sexual nature for the mainstream as their technology is transferred to profitable non-sexual purposes? In the future, non-sexual uses for human-resembling robots might include psychological therapy or companionship, and the future will reveal other uses commensurate with the capabilities that sex robots have acquired. Unlike most robotics industries, sex robots seek to provide an acceptable substitute for human companionship. Humanity is a long way from building robots that can be mistaken for humans, but given the goals of the AI development for sex robots, users may start ascribing them human features nonetheless.

## Risks Related to Sex Robots

Sex robots' ability to physically and emotionally resemble actual people—usually idealized and hyper-sexualized women—to provide gratifying intimate experiences is the key innovation and main risk driver. For example, a developer seeking to enhance the user experience with a sex robot may design a machine learning algorithm that builds trust between the human user and robot. Trust certainly benefits intimacy. But such a technology, once developed, may be deployed both within sex robots and other formats to induce user trust even potentially against the user's best interest. Advanced machine learning may allow robots to cultivate love and devotion, the ability to elicit personal information or to manipulate and influence behavior. These capabilities are all theoretically possible, and perhaps more importantly, they are profitable for AI sex robots to cultivate. With the worldwide sex technology reportedly worth 30 billion USD (Kleeman, 2017), the market may incentivize the development of AI capabilities that may be vastly more consequential than blinking silicon sex dolls.

Existing risks for sex toys have been documented. Noting the sharp increase of emergency room visits for sex toy-related injuries after 1999, Stabile (2013) appealed for regulation around sex toy labeling, safe design, and chemical testing. Conventional risks already demonstrated in sex toys include physical, biological and chemical, at levels that have compelled regulatory action in other product categories. For example, toxic phthalates that are banned in US children's toys due to risks of absorption through skin may comprise half the weight of some sex toys (Peters, 2006), which are designed for contact with sensitive and highly absorptive body parts. Inexpensive jelly-like materials also account for significant chemical hazards posed by phthalates that have been linked directly to organ damage and reproductive impacts. Physical injuries can occur through product misuse, malfunction, and lack of labeling or standardization in products. Biological hazards include infection from improper use, and from the use of inexpensive porous materials that can harbor microbes. Both physical and biological hazards are already widely documented through the National Electronic Injury Surveillance System database.

This hazard information is highly relevant for consumer risk because exposure to sex toys is significant throughout the US population, with nearly half of men and over half of all women reporting their use. Furthermore, injuries from sex toys are likely underreported due to associated stigma; indeed, multiple injuries became serious and even resulted in death due to the hesitance of consumers to seek treatment. The quantitative risk data do not align with the relative treatment of other product categories vs. sex toys: the National Electronic Injury Surveillance System for consumer products reports higher rates of injury from sex toys than for the last five products selected to be addressed via an Advanced Notice of Proposed Rule Making (ANPRM). No ANPRMs have been issued for sex toys. The unmet safety needs for sex toys may offer useful insight in navigating the complex landscape of risks represented within sex robots.

In addition to enhancing conventional risks, internet-enabled sex toys and sex robots also pose new cybersecurity risks that cannot be fully addressed under existing regulatory processes for other products. Such risks including lax security measures that allow uninvited parties to collect and store usage information or videos, or the potential hacking of remotely operated sex toys (Hern, 2016; Burgess, 2018; Devlin, 2018). With the expiration of the teledildonics patent in August 2018, the cybersex toy industry is now poised for rapid expansion, but without protections in place for consumers (Kobie, 2018). AI development for sex robots may increase consumer exposure to existing conventional risks and cybersecurity risks of sex toys (Table 1).

**Table 1:** Risks related to sex toys and sex robots.

	**Exposure**	**Hazards**	**Effects**
**Conventional risks**			
Physical	Sex toy usage ≈ 54% women (18–60) 45% men (18–60) (Stabile, 2013)	Design, electrification, mis-labeling of intended use	Physical injury
Biological		Microbial growth on porous surfaces or disease transfers through shared equipment	Infections
Chemical		Phthalates and other toxic materials, often newly developed or applied	Long term organ damage, endocrine disruption
**New risks**			
Cyber	Unknown subset of sex toy usage	Data collection, remote hacking	Privacy violations, blackmail opportunities
AI advancement	Unknown	Machine-learning to accomplish objectives of robot in a human form	Privacy violations, blackmail, manipulation, others

## The Government Needs to Protect Consumers

Existing regulations for sex toys fall under both the Consumer Product Safety Commission (CPSC) and the Food and Drug Administration (FDA). The CPSC oversees sex toy sales, but does not mandate or implement any labeling standards or testing, and the FDA has regulatory control over a small number of vibrators still archaically classified as obstetrical and gynecological medical devices. The remainder of sex toys, constituting the market's vast majority, are currently sold under the label “for novelty use only,” which exempts them from regulation or mandatory safety testing. As Stabile (2013) documents, consumers face risks from these toys, novelty use notwithstanding. Consumer risks from sex toys can be mitigated by subjecting them to federal standards as for other consumer products, but this has not yet been done.

Meanwhile, sexualized chatbots have already launched in several domains (Ellis, 2019). The stakes of unregulated AI may be much more far-reaching and perhaps analogous to the current controversies surrounding social media platforms' ability to shape users' moods and perspectives (Linkov et al., 2019). American society has already witnessed the capabilities of unseen algorithms to sow discord and polarization; how might this change when the algorithms stand before us in the form of idealized humans? Bascandziev and Harris (2014) found that pre-school children given conflicting information from two people tend to trust the more attractive person. Will this relationship hold when the attractive person is made of silicon, or appears as a virtual persona on a platform like Instagram?

More data are needed to characterize risks for sex robots and their AI capabilities, but government agencies must actively engage in gathering, analyzing, and acting upon the data. The drivers of the privacy shroud for sex-based technologies (e.g., misogynistic or religion-influenced views of normative sexuality) should be examined and in some cases challenged in order to forecast and responsibly manage future risks. AI innovation of sex robots may further expose people to the harms of sex toys while spurring the development of machine learning products. Regulators need to place this topic in the light and examine it with the same professional dispassionate objectivity that is granted to other consumer products.

## Author Contributions

IL and CH formulated the problem statement for the paper, and SG gathered sources and conducted the research under guidance from CH, BT, and SG framed the argument, and SG drafted the paper, which was revised and finalized by CH, BT, and IL.

### Conflict of Interest

The authors declare that the research was conducted in the absence of any commercial or financial relationships that could be construed as a potential conflict of interest.
